# Biochemical and Proteomic Characterization of Alkaptonuric Chondrocytes

**DOI:** 10.1002/jcp.24033

**Published:** 2011-12-29

**Authors:** Daniela Braconi, Giulia Bernardini, Claretta Bianchini, Marcella Laschi, Lia Millucci, Loredana Amato, Laura Tinti, Tommaso Serchi, Federico Chellini, Adriano Spreafico, Annalisa Santucci

**Affiliations:** 1 Dipartimento di Biotecnologie, Università degli Studi di SienaSiena, Italy; 2 Centro Interdipartimentale per lo Studio Biochimico delle Patologie Osteoarticolari, Università degli Studi di SienaSiena, Italy; 3 Sezione di Reumatologia, Dipartimento di Medicina Clinica e Scienze Immunologiche, Università degli Studi di SienaPoliclinico Le Scotte, Siena, Italy

## Abstract

Alkaptonuria (AKU) is a rare genetic disease associated with the accumulation of homogentisic acid (HGA) and its oxidized/polymerized products which leads to the deposition of melanin-like pigments (ochronosis) in connective tissues. Although numerous case reports have described ochronosis in joints, little is known on the molecular mechanisms leading to such a phenomenon. For this reason, we characterized biochemically chondrocytes isolated from the ochronotic cartilage of AKU patients. Based on the macroscopic appearance of the ochronotic cartilage, two sub-populations were identified: cells coming from the black portion of the cartilage were referred to as “black” AKU chondrocytes, while those coming from the white portion were referred to as “white” AKU chondrocytes. Notably, both AKU chondrocytic types were characterized by increased apoptosis, NO release, and levels of pro-inflammatory cytokines. Transmission electron microscopy also revealed that intracellular ochronotic pigment deposition was common to both “white” and “black” AKU cells. We then undertook a proteomic and redox-proteomic analysis of AKU chondrocytes which revealed profound alterations in the levels of proteins involved in cell defence, protein folding, and cell organization. An increased post-translational oxidation of proteins, which also involved high molecular weight protein aggregates, was found to be particularly relevant in “black” AKU chondrocytes. J. Cell. Physiol. 227: 3333–3343, 2012. © 2011 Wiley Periodicals, Inc.

Alkaptonuria (AKU; MIM no. 203500) is an autosomal recessive inherited rare disease resulting from a deficiency of the enzyme homogentisate 1,2-dioxygenase (HGO; EC 1.13.11.) that normally splits the aromatic ring of homogentisic acid (HGA; 2,5-dihydroxyphenylacetic acid), an intermediary product of the catabolism of tyrosine and phenylalanine (La Du et al., 1958; Fernandez-Canon et al., 1996; Phornphutkul et al., 2002). As a consequence, HGA is not further metabolized and is excreted with urine, to which it imparts a black discoloration upon standing due to its oxidation to benzoquinone acetic acid (BQA) (Zannoni et al., 1962). The same oxidation occurs within the body, where HGA is accumulated, allowing the formation of oxidized/polymerized melanin-like products imparting to connective tissues (especially skin, cardiovascular system, and joints) a characteristic pathologic pigmentation known as “ochronosis” (Zannoni et al., 1962; O'Brien et al., 1963; Phornphutkul et al., 2002; Helliwell et al., 2008). Ochronosis leads to rapid degeneration and inflammation of joints where a concomitant musculoskeletal involvement might induce a severe and sometimes crippling form of arthropathy (Selvi et al., 2000; Helliwell et al., 2008).

Yet well described from the clinical point of view, alkaptonuric ochronosis and its molecular mechanisms have not been explored to any significant degree due mainly to the rarity of the disease [AKU has a 1:250,000–1,000,000 incidence (Tinti et al., 2011a)] as well as to the lack of suitable models to study it. We recently introduced novel human ochronotic cell and serum models in which we demonstrated how HGA induces protein oxidation and aggregation (Braconi et al., 2010a, b; Tinti et al., 2010, 2011b). In the present paper, we provided the first proteomic characterization of AKU chondrocytes, obtained from the ochronotic cartilage of AKU patients. Our findings indicated that AKU cells experience inflammatory stimuli and are characterized by relevant alterations in the expression of proteins involved in cell defence, protein folding and cell organization, sharing similarities with osteoarthritis (OA). Additionally, AKU cells experience significant protein oxidation and aggregation which might help, in turn, the production of ochronotic pigments.

## Materials and Methods

### Reagents

Unless otherwise indicated, all high quality reagents and antibodies were from Sigma–Aldrich (St. Louis, MO). All water used was Milli-Q (Millipore, Bedford, MA).

### Isolation and culture of human chondrocytes from ochronotic AKU patients

AKU chondrocytes were obtained, after informed consent in accordance with the Declaration of Helsinki, from hip cartilage fragments of three alkaptonuric patients suffering from ochronotic arthropathy and undergone surgery for hip replacement. The study received approval from the Local Ethics Committee. The characteristics of patients enrolled for the study are schematically reported below:

Patient #1: female, age 62, 4/4 backbone impairment, 4/4 articular joints impairment, two orthopaedic surgical interventions, urinary HGA level (24 h): 300 mg/dl.
Patient #2: male, age 60, 4/4 backbone impairment, 4/4 articular joints impairment, five orthopaedic surgical interventions, urinary HGA level (24 h): 371 mg/dl.
Patient #3: female, age 69, 4/4 backbone impairment, 4/4 articular joints impairment, two orthopaedic surgical interventions, urinary HGA level (24 h): 475 mg/dl.

At visual inspection, donors' cartilages presented both a white and dark coloration and we obtained, from separate digestion of these different fragments, two cell populations that we named “white” and “black” chondrocytes. Immediately after surgery, cartilage was cut aseptically and minced in small pieces. Fragments were washed in Dulbecco's modified Eagle's medium (DMEM) containing 2% penicillin/streptomycin solution and 0.2% amphoterycin B. Chondrocytes were isolated by sequential enzymatic digestion: 30 min with 0.1% hyaluronidase, 1 h with 0.5% pronase, and 1 h with 0.2% collagenase at 37 °C in wash solution (DMEM + penicillin/streptomycin solution + amphoterycin B). The cells suspension was then filtered twice using 70 µm nylon meshes, washed and centrifuged for 10 min at 700×*g*. Cell pellets were then re-suspended in DMEM containing 10% fetal calf serum (FCS) and 0.33 mM HGA and expanded in monolayer culture at 37°C in 95% relative humidity and in a 5% CO_2_ atmosphere. HGA was added to mimic in vitro the AKU pathological condition; the concentration used, 0.33 mM, is in the range of circulating HGA in AKU patients' serum (Angeles et al., 1989) and it has been successfully used in several AKU models that we have set up and characterized previously (Braconi et al., 2010a, b; Tinti et al., 2010, 2011a, b). In parallel, control (non-AKU) chondrocytes from OA samples were obtained and cultured under the same conditions but avoiding HGA addition.

### Cell proliferation

The DNA content of cell pellets from each culture was used as a parameter of cell proliferation. DNA was extracted with a high pure PCR template preparation kit according to manufacturer's instructions (Roche Diagnostics, Mannheim, Germany) and quantified with Qubit™ (Invitrogen, Grand Island, NY).

### Apoptosis

Apoptosis was analyzed by flow cytometry evaluating Annexin V binding (DAKO, Milano, Italy). Cells were stained with fluorescein isothiocyanate (FITC)-Annexin V and propidium iodide.

### NO release

Nitrite levels were measured in chondrocyte culture supernatants with Griess reagent (1% sulfanilamide, 0.1% napthylenediamine dihydrochloride, and 2.5% phosphoric acid) (Rediske et al., 1994). Equals volumes (100 µl) of supernatant and Griess reagent were incubated in microtitre plates at room temperature for 5 min. Nitrite concentrations were determined spectrophotometrically at 550 nm using a standard curve generated with serial dilutions of sodium nitrite.

### Transmission electron microscopy (TEM)

Once isolated from ochronotic specimens, AKU chondrocytes were fixed for 2 h at 4°C in cold Karnovsky fixative, rinsed overnight in 0.1 M pH 7.2 cacodylate buffer, post-fixed for 1 h at 4°C in 1% buffered OsO_4_, dehydrated in a graded series of ethanol, and embedded in Epon-Araldite. Ultrathin sections cut with an LKB III ultramicrotome were mounted on copper grids, stained with uranyl acetate and lead citrate and then photographed with a Philips CM10 electron microscope. At least 100 cells from each group were observed.

### Cytokine assay

The release of IL-1β, IL-6 IL-8, IL-10, and TNFα from AKU chondrocytes was evaluated in cell culture supernatant by means of multiplex assay for cytokine quantification (Bioplex, Bio-Rad, Hercules, CA). Cytokine concentrations were calculated using a standard curve established from serial dilutions of each cytokine standard as described in the manufacturer's protocol and expressed as picograms per milliliter of culture medium.

### Two-dimensional electrophoresis (2D-PAGE) and Western blot

Cells were washed twice with sterile PBS and resuspended in 50 µl of a buffer containing 65 mM DTE, 65 mM CHAPS, 9 M urea, and 35 mM Tris-base. Cell disruption was achieved by sonicating briefly in an ice bath. Protein content in cell lysates was assessed according to Bradford (Bradford, 1976).

Cell lysates were first mixed with a buffer containing 8 M urea, 35 mM CHAPS, 10 mM DTE, and a trace of bromophenol blue. One hundred micrograms [2D gels to be transferred onto nitrocellulose (NC) membranes] or 50 µg (2D gels to be silver stained) of proteins were adsorbed onto Immobiline Dry Strips (IPG 18 cm, nonlinear 3–10 pH range, Bio-Rad) for 10 h. Isoelectric focusing (IEF) was carried out with a Protean IEF cell (Bio-Rad). The voltage was linearly increased from 300 to 3,500 V during the first 3 h and then stabilized at 5,000 V for 22 h (total 110 kV × h).

Prior to sodium dodecyl sulfate–polyacrylamide gel electrophoresis (SDS–PAGE), IPG were equilibrated in 6 M urea, 30% (v/v) glycerol, 2% (w/v) SDS, 0.05 M Tris–HCl pH 6.8 containing first 2% (w/v) DTE, and later 2.5% (w/v) iodoacetamide (IAA). SDS–PAGE was carried out applying 40 V per gel until the dye front reached the bottom of gels.

Silver ammoniacal staining was carried out according to Switzer (Switzer et al., 1979) whereas gels to be transferred onto NC were washed and equilibrated in a transfer buffer [50 mM Tris, 40 mM glycine, 1.3 mM SDS, 20%, v/v methanol] and protein transfer was carried out using a semidry Novablot transblot cell (Bio-Rad) applying 0.7 mA/cm^2^ for 75 min. Protein transfer was checked by staining with 0.2% Ponceau S in 3% (v/v) TFA for 3 min and de-staining with water.

For the Western blot analysis of carbonylated proteins, after the IEF IPG strips were briefly rinsed with water and incubated at room temperature for 20 min with 10 mM 2,4-dinitrophenylhydrazine (DNPH) in 5% (v/v) TFA to allow derivatization of protein carbonyls (Reinheckel et al., 2000). Strips were washed twice with a solution containing 8 M urea, 20% (v/v) glycerol, 9 M SDS, and 150 mM Tris–HCl pH 6.8. For the immunorevelation of protein carbonyls, NC sheets were incubated with rabbit anti-dinitrophenyl antibodies 1:10,000 (over-night at 4°C), followed by peroxidase-conjugated anti-rabbit antibodies 1:7,000 (2 h at room temperature) and revelation was achieved through chemiluminescence (Immu-Star HRP, Bio-Rad).

Protein spots of interest were identified by MALDI-TOF mass spectrometry (MS) or by gel matching with proteomic reference maps already produced, calibrated, and characterized in our laboratories (Spreafico et al., 2006, 2009).

### Image analysis

Images of gels and films were acquired (Image Scanner, Amersham Biosciences, Uppsala, Sweden) and analyzed with Image Master™ Platinum (Amersham Biosciences). For comparative proteomic analysis, spot% relative abundance was adopted; for multiple spots identified as different molecular species of a same protein, the average% relative abundance was calculated. For the quantitative analysis of immunorevealed protein carbonyls, the intensity of bands, which is automatically normalized by Image Master™ Platinum against the surrounding background, was chosen as the reference parameter.

### Statistical analysis

All of the experiments were carried out in triplicate; data are presented as average values with standard deviation. Student's *t*-test and multiple-measurement ANOVA analysis followed by the Bonferroni-type multiple comparison were used when necessary. Differences with at least a *P*-value ≤0.05 were considered significant. For comparative proteomic and redox proteomic analyses, only representative gels and films are shown.

## Results

**Fig. 1:**
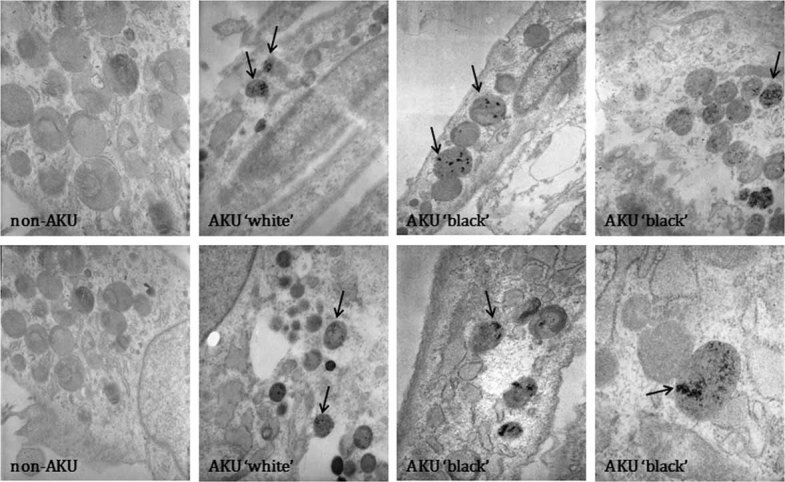
Deposition of ochronotic pigments, detected by TEM, in “white” and “black” AKU chondrocytes and their controls (non-AKU). Arrows indicate the presence of ochronotic pigments. Only representative images are shown.

In this work, we characterized two distinct types of primary chondrocytes (namely “white” and “black” AKU chondrocytes) obtained from AKU patients suffering from ochronotic arthropathy who underwent surgery. TEM analysis revealed that both “white” and “black” AKU chondrocytes contained intracellular ochronotic pigments (Fig. 1) and showed a similar morphology. The finding of pigments in “white” chondrocytes is particularly noteworthy, because it demonstrated that deposition of ochronotic pigments occurred even in areas where no macroscopic evidence of pigmentation was observed (Kutty et al., 1973). Therefore, intracellular pigment deposition possibly precedes deposition in the extracellular matrix. Another noteworthy feature was the presence of ochronotic deposits of varying shapes and sizes also within some vacuoles (Fig. 1).

**Fig. 2:**
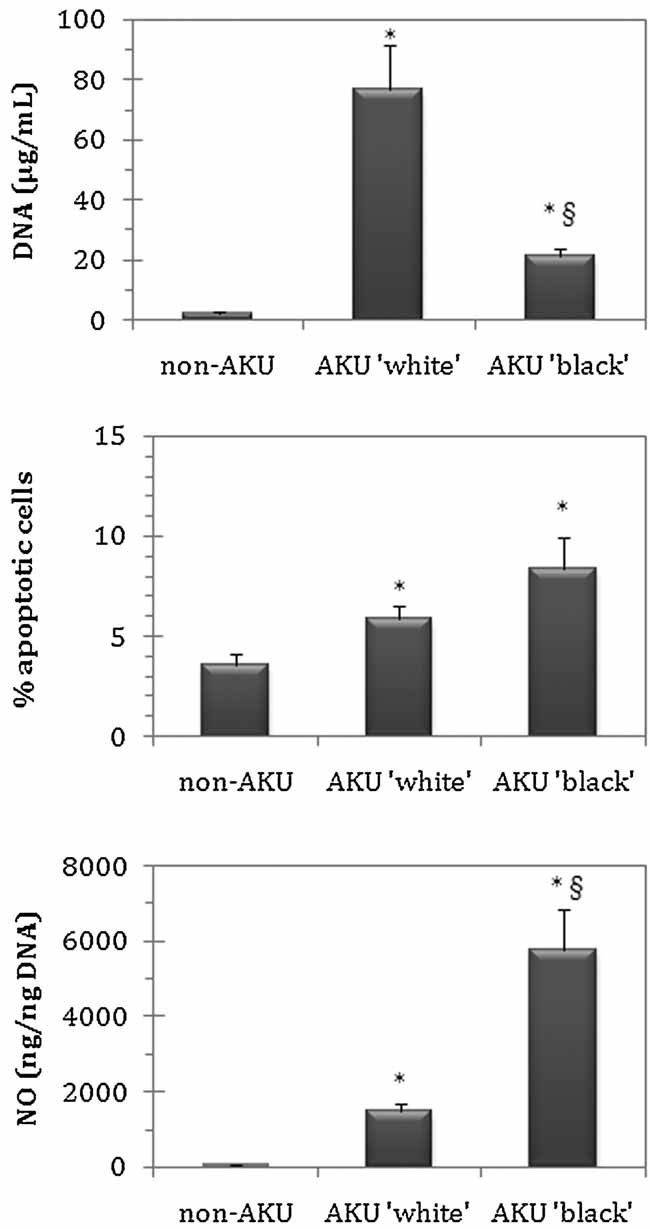
Cell proliferation (A), apoptosis (B), and NO release (C) in “white” and “black” AKU chondrocytes and their control (non-AKU). Cell proliferation was assayed by measuring the DNA content of cell pellets; apoptosis was assayed by Annexin V-FITC/propidium iodide staining and flow cytometry; NO release in culture supernatants was assayed by Griess reagent, as detailed under Materials and Methods Section. Experiments were performed in triplicate; data are presented as average values with standard deviation. Statistical significance compared to non-AKU control (*P ≤ 0.05) and between “white” and “black” AKU chondrocytes (§*P* ≤ 0.05) is indicated.

Both types of AKU cells showed a proliferation rate higher than the control (+30.8- and +8.9-fold-change for “white” and “black” chondrocytes, respectively) and “white” AKU chondrocytes had a proliferation rate higher than “black” ones (with a +6.1-fold change) (Fig. 2A). Since exposure to HGA may induce cytotoxicity (Kirkpatrick et al., 1984; Angeles et al., 1989), we investigated also cell apoptosis and found that “white” and “black” chondrocytic cultures were both characterized by a higher% of apoptotic cells when compared to the control (+1.5- and +2.0-fold-change, respectively) (Fig. 2B). Interestingly, the release of NO in AKU chondrocytes was significantly higher than in the control. Particularly, +33- and +126-fold-change values were recorded for NO levels in “white” and “black” chondrocytes, respectively (Fig. 2C).

### Cytokine expression

**Fig. 3:**
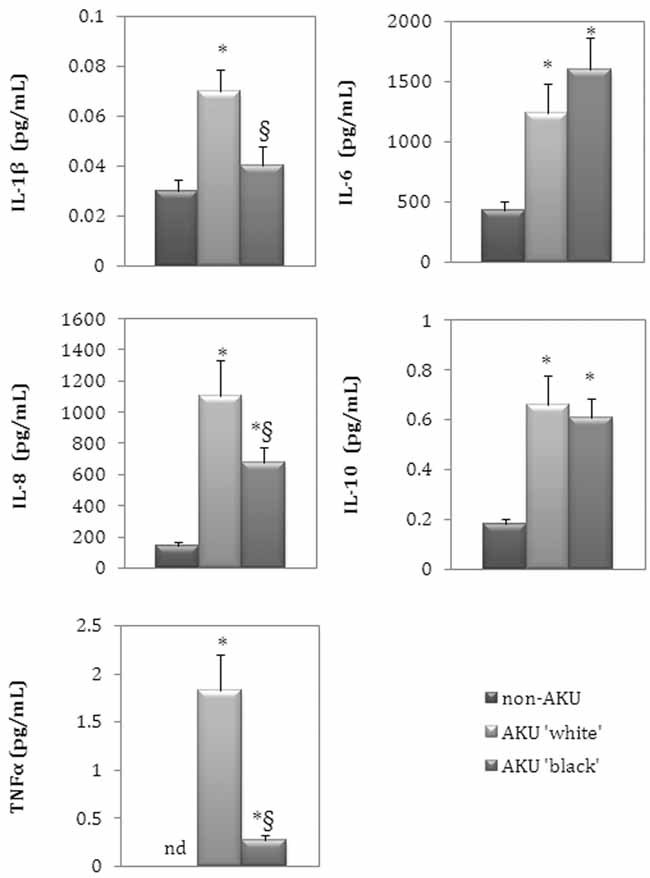
Release of cytokines in “white” and “black” AKU chondrocytes and their control (non-AKU) by means of multiplex assay, as detailed under Materials and Methods Section. Experiments were performed in triplicate; data are presented as average values with standard deviation. Statistical significance compared to non-AKU control (*P ≤ 0.05) and between “white” and “black” AKU chondrocytes (§*P* ≤ 0.05) is indicated. nd: non-detectable.

To evaluate the inflammatory status of AKU ochronotic chondrocytes, we measured the levels of IL-1β, IL-6, IL-8, IL-10, and TNFα in cell culture supernatants. With the only exception of IL-1β in “black” AKU chondrocytes, which did not show any difference with respect to the control, for each assayed cytokine we found significantly higher levels in AKU cells, as shown in Figure 3. Interestingly, levels of both IL-1β and TNFα were significantly higher in “white” AKU chondrocytes, whereas “black” chondrocytes showed only a significant increase in TNFα levels. Upon injuries or diseases, cytokines such as IL-1β and TNFα can be found as major mediators of inflammation within articular cartilages undergoing repair/regeneration (Pelletier et al., 2001; Kobayashi et al., 2005; Wehling et al., 2009). Both cytokines induce an imbalance towards the catabolic pathway (Fernandes et al., 2002) through up-regulation of metalloproteinases (MMPs), aggrecanases, inducible NO synthase, and cyclooxygenase 2 (Umlauf et al., 2010). Their pro-inflammatory action is also mediated by an enhanced production of IL-6 and IL-8 (Umlauf et al., 2010) together with induction of apoptosis (Fischer et al., 2000; Aizawa et al., 2001; Aigner and Kim, 2002). Though players in a very complex scenario, IL-1β and TNFα are associated with cartilage degradation and synovial inflammation observed in OA (Fernandes et al., 2002).

IL-6 levels were significantly increased in AKU “white” and “black” chondrocytes with respect to the control (+2.9- and +3.7-fold-change, respectively), similarly to what we observed for IL-8 levels (+7.7-fold-change in “white” and +4.7-fold-change in “black” AKU chondrocytes). IL-6 is a co-factor of the catabolic effects of IL-1β (Flannery et al., 2000) enhancing the production of MMP-13 and, consequently, the IL-1β-induced degradation of proteoglycan in cartilage (Honorati et al., 2002). IL-8 belongs to the family of chemokines. Recently, a key role for the chondrocytic chemokine/chemokine receptor system in OA has been suggested (Facchini et al., 2005). Notably, IL-8 was used as a biomarker for rheumatoid arthritis on the basis of its elevated levels found in synovial fluid of affected patients (Kokebie et al., 2011).

IL-10 levels were 3.7 and 3.4 times higher in AKU “white” and “black” chondrocytes than in the control, respectively. In OA, cartilage and synovium show increased levels of IL-10 (Isomaki and Punnonen, 1997; Jorgensen et al., 1998; Moo et al., 2001) that are thought to inhibit the synthesis of other pro-inflammatory cytokines (Katsikis et al., 1994; Lechman et al., 1999). Nevertheless, the exact roles of IL-10 in inflammatory diseases, and especially in chondrocyte homeostasis, need to be fully realized. In particular, the study of the interplay between IL-10 and TNFα revealed that, if on the one hand IL-10 can strongly inhibit the TNFα-induced down regulation of aggrecan expression, on the other it can only have minor effects in contrasting the TNFα-induced collagen type II suppression (Muller et al., 2008).

### Comparative proteomics

**Fig. 4:**
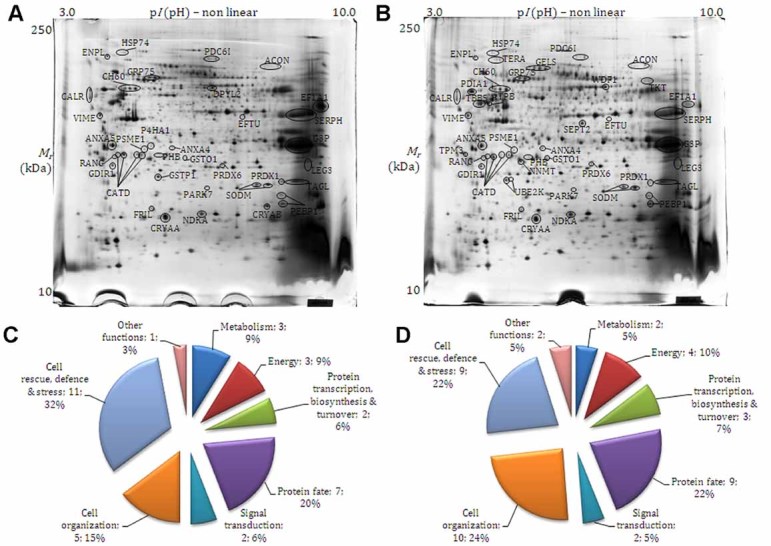
Proteomic analysis. Silver stained 2D maps of “white” (A) and “black” (B) AKU chondrocytes. The comparative analysis carried out against a non-AKU chondrocytes control map (not shown) allowed the identification of differently expressed proteins (fold change at least 2 in protein% relative abundance) that are indicated with their abbreviated name. Functional classification of differently expressed proteins is indicated in (C) and (D) for “white” and “black” AKU chondrocytes, respectively. Representative images from a triplicate set are shown.

Silver staining allowed to reveal nearly 3,000 spots in “white” and “black” AKU chondrocyte proteomic maps (Fig. 4A, B). MALDI-ToF MS and gel matching with reference maps (Spreafico et al., 2006, 2009) allowed the identification of 78 gene products. Once set a statistically significant threshold ≥2.0 for fold-change values in protein relative abundance ratio, the quantitative analysis against the control proteomic map revealed that 34 and 41 proteins were differently expressed in “white” and “black” AKU chondrocytes, respectively. The description of differently expressed proteins and fold-change values are schematically reported in Table I (see also Supplementary Material). The functional classification of differently expressed proteins is reported in Figure 4C, D. The protein repertoires of “white” and “black” AKU chondrocytes were quite similar and the most significant alterations with respect to the control were found for proteins involved in:

- *Protein fate*: Twenty percent and 22% of proteins differently expressed in AKU “white” and “black” chondrocytes, respectively. We generally found an under expression of identified proteins involved in folding, maturation, and transport of proteins. It is noteworthy to mention the under-expression in “black” AKU chondrocytes of protein disulfide-isomerase (PDIA1), that is thought to contribute to the structural integrity of cartilage tissue, allowing a suitable response of load bearing joints to mechanical stress (Grimmer et al., 2006). We observed also an over-expression of programmed cell death 6-interacting protein (PDC6I) in both “white” and “black” AKU chondrocytes. Since PDC6I participates in the physiological sorting pathway for tyrosinase, a key enzyme in melanogenesis (Theos et al., 2005) an involvement of PDC6I in the production of ochronotic pigment in AKU at the cartilage level can be hypothesized.
- *Cell structure and organization*: Fifteen percent and 24% of proteins differently expressed in AKU “white” and “black” chondrocytes, respectively. Changes in the organization and distribution of structural proteins characterize various pathologies, including OA (Fioravanti et al., 2003; Capin-Gutierrez et al., 2004; Holloway et al., 2004). Levels of several structural proteins were lower in AKU chondrocytes with respect to the control, such as vimentin (VIME), under-expressed in both “white” and “black” AKU chondrocytes, and gelsolin (GELS), under-expressed in “black” chondrocytes. VIME is the most sensitive sensor for mechanically induced stress, being necessary to maintain chondrocyte stiffness and allow a proper mechanotransduction (Li et al., 2010). GELS, regulating chondrocyte architecture and cell-matrix interactions, has been indicated as a prognostic and diagnostic marker of OA (De Ceuninck et al., 2005). Together, VIME and GELS contribute together to synovial fluid functions (Gobezie et al., 2007).
- *Cell rescue, defence, and stress response*: Thirty-two percent and 22% of proteins differently expressed in AKU “white” and “black” chondrocytes, respectively. AKU chondrocytes showed altered levels of proteins fundamental for the protection from oxidative stress, like previously indicated by our group in a chondrocytic line used as a model of AKU (Braconi et al., 2010b). Though alpha-crystallin A chain (CRYAA), peroxiredoxin-1 (PRDX1), peroxiredoxin-6 (PRDX6), and serpin H1 (SERPH) were over-expressed both in “white” and “black” AKU chondrocytes compared to the control, lower protein levels were found in “white” and “black” AKU chondrocytes for most of the identified proteins. This is the case of the mitochondrial 60 kDa HSP (CH60), alpha-crystallin B chain (CRYAB), endoplasmin (ENPL), glutathione *S*-transferase omega-1 (GSTO1), glutathione *S*-transferase P (GSTP1), heat shock 70 kDa protein 4 (HSP74), and mitochondrial superoxide dismutase (SODM). The simultaneous deficiency of these proteins reinforce the hypothesis that AKU cells cannot adequately control reactive oxygen species (ROS)- and quinone-mediated toxicity.

**Table I:** Comparative proteomics of AKU chondrocytes

Spot	AN	Gene	Protein	Biological processes^a^	loc.^b^	CHW/CTR^c^	CHB/CTR^c^	CHB/CHW	ID^d^
Metabolism									
Nucleotide, nucleoside, and nucleic acids									
DPYL2	Q16555	*DPYSL2*	Dihydropyrimidinase-related protein 2	Signal transduction. Necessary for remodeling of the cytoskeleton	C, Cs	−2.8			GM
NDKA	P15531	*NME1*	Nucleoside diphosphate kinase A	Nucleotide metabolism, cell differentiation, and regulation of apoptosis	C, N	+2.1	+2.3		GM
PHB	P35232	*PHB*	Prohibitin	Inhibition of DNA synthesis, regulation of proliferation	Mt	−11.9	−3.2	+3.7	GM
Energy									
Carbohydrate metabolism									
ACON	Q99798	*ACO2*	Aconitate hydratase, mitochondrial	Tricarboxylic acid cycle; isocitrate from oxaloacetate: step 2/2	Mt, N	+3.7	+2.8		GM
G3P	P04406	*GAPDH*	Glyceraldehyde-3-phosphate dehydrogenase	Glycolysis (pyruvate from d-glyceraldehyde 3-phosphate: step 1/5). Independent of its glycolytic activity, it is also involved in membrane trafficking in the early secretory pathway and in oxido- reductase reactions	C, M	−5.2	−3.9		MS
Respiration and fermentation									
ATPB	P06576	*ATP5B*	ATP synthase subunit beta, mitochondrial	ATP synthesis, regulation of intracellular pH	Mt		−3.8	−2.5	GM
LDHB	P07195	*LDHB*	l-lactate dehydrogenase B chain	Fermentation; pyruvate fermentation to lactate; (S)-lactate from pyruvate: step 1/1	C	−2.5	—	—	GM
Pentose-phosphate pathway									
TKT	P29401	*TKT*	Transketolase	Sedoheptulose 7-phosphate + d-glyceraldehyde 3-phosphate = d-ribose 5-phosphate + d-xylulose 5-phosphate; response to oxidative stress	C		+2.2	+2.0	GM
Transcription, synthesis, and turnover of proteins									
CATD	P07339	*CTSD*	Cathepsin D	Acid protease active in intracellular protein breakdown	Lys, Mel, S	−4.7	−3.8		MS
EF1A1	P68104	*EEF1A1*	Elongation factor 1-alpha 1	Protein biosynthesis	C		−9.6	−10.1	GM
EFTU	P49411	*TUFM*	Elongation factor Tu, mitochondrial	Protein biosynthesis	Mt	−6.4	−7.5		GM
Protein fate (folding, maturation, and transport)									
CALR	P27797	*CALR*	Calreticulin	Molecular calcium-binding chaperone promoting folding, oligomeric assembly and quality control in the ER	C, ER, EM, S	−2.3	−2.9		GM
GRP75	P38646	*HSPA9*	75 kDa glucose-regulated protein	Control of cell proliferation and cellular aging, may also act as a chaperone; has anti-apoptotic functions	Mt	−2.0	−2.1		GM
P4HA1	P13674	*P4HA1*	Prolyl 4-hydroxylase subunit alpha-1	Catalyzes the post-translational formation of 4-hydroxyproline in -Xaa-Pro-Gly- sequences in collagens and other proteins; it is involved in redox reactions		−2.2			GM
PARK7	Q99497	*PARK7*	Protein DJ-1	May function as a redox-sensitive chaperone and as a sensor for oxidative stress; prevents aggregation of alpha-synuclein	C, N, may be associated to Mt after oxidative stress	−3.3	−3.4		GM
PDC6I	Q8WUM4	*PDCD6IP*	Programmed cell death 6-interacting protein	Protein transport; may play a role in the regulation of both apoptosis and cell proliferation	C, Cs, Mel	+12.8	+8.2	−2.2	GM
PDIA1	P07237	*P4HB*	Protein disulfide-isomerase	Catalyzes the formation, breakage, and rearrangement of disulfide bonds; at the cell surface, seems to act as a reductase that cleaves disulfide bonds of proteins attached to the cell. At high concentrations, functions as a chaperone that inhibits aggregation of misfolded proteins; at low concentrations, facilitates aggregation (anti-chaperone activity)	ER, Mel, M		−2.3		GM
PEBP1	P30086	*PEBP1*	Phosphatidylethanolamine-binding protein 1	Phosphatidylethanolamine-binding protein; serine protease inhibitor	C	+16.4	+27.2		GM
PSME1	Q06323	*PSME1*	Proteasome activator complex subunit 1	Implicated in immunoproteasome assembly and required for efficient antigen processing	C, Pr	−3.2	−2.4		GM
TERA	P55072	*VCP*	Transitional endoplasmic reticulum ATPase	ER-associated protein catabolic process, ER-unfolded protein response, protein ubiquitination, retrograde protein transport (ER to cytosol)	C, N		−2.9		GM
UBE2K	P61086	*UBE2K*	Ubiquitin-conjugating enzyme E2 K	Protein ubiquitination	C		−2.6		GM
Signal transduction									
GDIR1	P52565	*ARHGDIA*	Rho GDP-dissociation inhibitor 1	Regulates the GDP/GTP exchange reaction of the Rho proteins	C	−3.5	−3.5		GM
RANG	P43487	*RANBP1*	Ran-specific GTPase-activating protein	Inhibits GTP exchange on Ran; may act in an intracellular signaling pathway which may control the progression through the cell cycle by regulating the transport of protein and nucleic acids across the nuclear membrane	C, N	−13.5	−16.6		GM
Cellular organization									
Cytoskeleton and microtubules									
GELS	P06396	*GSN*	Gelsolin	Binds to actin and to fibronectin preventing monomer exchange and promoting the assembly of monomers into filaments (nucleation) as well as sever filaments already formed. Defects in GSN are the cause of amyloidosis type 5 (AMYL5) [MIM:105120]; also known as familial amyloidosis Finnish type. AMYL5 is a hereditary generalized amyloidosis due to gelsolin amyloid deposition. It is typically characterized by cranial neuropathy and lattice corneal dystrophy. Most patients have modest involvement of internal organs, but severe systemic disease can develop in some individuals causing peripheral polyneuropathy, amyloid cardiomyopathy, and nephrotic syndrome leading to renal failure	C, Cs, S, amyloid		−2.0	−2.2	GM
TAGL	Q01995	*TAGLN*	Transgelin	Actin cross-linking/gelling protein. Involved in calcium interactions and contractile properties of the cell that may contribute to replicative senescence	C	+9.2	+17.0		MS
TBB5	P07437	*TUBB*	Tubulin beta chain	Major constituent of microtubules	C		−2.9		GM
Cell cycle									
SEPT2	Q15019	*SEPT2*	Septin-2	Cell division, mitosis	C		+2.0		GM
Annexin family									
ANXA4	P09525	*ANXA4*	Annexin A4	Calcium/phospholipid-binding protein which promotes membrane fusion and is involved in exocytosis; anti-apoptosis		−4.9	−2.4	+2.0	GM
ANXA5	P08758	*ANXA5*	Annexin A5	Calcium-regulated membrane-binding protein, anti-apoptosis	C, N, M	−2.3	−2.6		GM
Intermediate filaments family									
VIME	P08670	*VIM*	Vimentin	Class-III intermediate filaments found in various non-epithelial cells	C	−5.2	−4.1		GM
Other functions									
LEG3	P17931	*LGALS3*	Galectin-3	Cell differentiation	N, C, M	−3.3	−11.3	−3.4	GM
TPM3	P06753	*TPM3*	Tropomyosin alpha-3 chain	Stabilization of cytoskeleton and actin filaments, cell motion	C, Cs		−2.4		GM
WDF1	Q8IWB7	*WDFY1*	WD repeat and FYVE domain-containing protein 1	Phosphatidylinositol binding	C, N		−2.6		GM
Cell rescue, defence, and stress									
CH60	P10809	*HSP60*	60 kDa heat shock protein, mitochondrial	Implicated in mitochondrial protein import and macromolecular assembly, may facilitate the correct folding of imported proteins, prevent misfolding and promote the refolding and proper assembly of unfolded polypeptides generated under stress conditions in the mitochondrial matrix	Mt	−2.4	−2.7		GM
CRYAA	P02489	*CRYAA*	Alpha-crystallin A chain (Heat shock protein beta-4)	Anti-apoptosis, protein folding, unfolded protein binding, response to heat	C	+2.7	+2.5		GM
CRYAB	P02511	*CRYAB*	Alpha-crystallin B chain	Anti-apoptosis, protein folding, unfolded protein binding, response to heat	**—**	**−2.2**			MS
ENPL	P14625	*HSP90B1*	Endoplasmin	Molecular chaperone that functions in the processing and transport of secreted proteins. Functions in endoplasmic reticulum associated degradation (ERAD). Has ATPase activity	C, ER, Mel	−3.6	−4.8		GM
				Plays a role in protein folding and transport; has anti-apoptotic functions; response to hypoxia					
GSTO1	P78417	*GSTO1*	Glutathione *S*-transferase omega-1	Exhibits glutathione-dependent thiol transferase and dehydroascorbate reductase activities	C	−5.2	−2.2	+2.3	GM
				RX + glutathione = HX + R-*S*-glutathione					
GSTP1	P09211	*GSTP1*	Glutathione *S*-transferase P	Conjugation of reduced glutathione to a wide number of exogenous and endogenous hydrophobic electrophiles. It can have anti-apoptotic functions	C	−2.6			MS
HSP74	P34932	*HSPA4*	Heat shock 70 kDa protein 4	Stress response; play a role in the unfolded protein response	C, N	−2.8	−5.4		GM
PRDX1	Q06830	*PRDX1*	Peroxiredoxin-1	Cell redox-homeostasis, peroxide catabolic process, and cell proliferation	C, N, Mel	+7.6	+8.2		GM
PRDX6	P30041	*PRDX6*	Peroxiredoxin-6	Cell redox homeostasis and protection against oxidative injury	C, N, L	P	P		GM
SERPH	P50454	*SERPINH1*	Serpin H1	Chaperone in the biosynthetic pathway of collage, it is induced by heat shock and plays a role in the response to unfolded protein	C	+2.7	+3.1		GM
SODM	P04179	*SOD2*	Superoxide dismutase [Mn], mitochondrial	Destroys radicals which are normally produced within the cells and which are toxic to biological systems 2 superoxide + 2 H^+^ = O_2_ + H_2_O_2_	Mt	−5.7	−4.5		GM
Other functions									
FRIL	P02792	*FTL*	Ferritin light chain	Iron storage and homeostasis, involved in redox reactions	R	+2.0	+2.1		GM
NNMT	P40261	*NNMT*	Nicotinamide *N*-methyltransferase	Catalyzes the *N*-methylation of nicotinamide and other pyridines to form pyridinium ions (this activity is important for biotransformation of many drugs and xenobiotic compounds)	C		−3.2	−3.4	GM

AN, accession number.

Proteins whose levels are altered in AKU “white” (CHW) and “black” (CHB) chondrocytes with respect to non-AKU control (CTR) chondrocytes.

a Protein biological processes.

b Protein sub-cellular localization: C (cytosol); Cs (cytoskeleton); CJ (cell junction); M (membrane); BM (basal membrane); EM (extracellular matrix); Mel (melanosome); Mt (mitochondrion); N (nucleus); Pe (peroxisome); Pr (proteasome); ER (endoplasmic reticulum); S (secreted); L (lysosome). Retrieved by UniProt knowledgebase (http://www.uniprot.org/).

c Fold-change in protein% relative abundance (as average values in case of multiple spots); (+) over-expressed proteins, (−) under-expressed protein according to the ratio indicated. “A” and “P” indicate the absence or presence of proteins, respectively.

d Identification method (GM: gel-matching; MS: MALDI-TOF mass spectrometry).

### Redox-proteomics

**Fig. 5:**
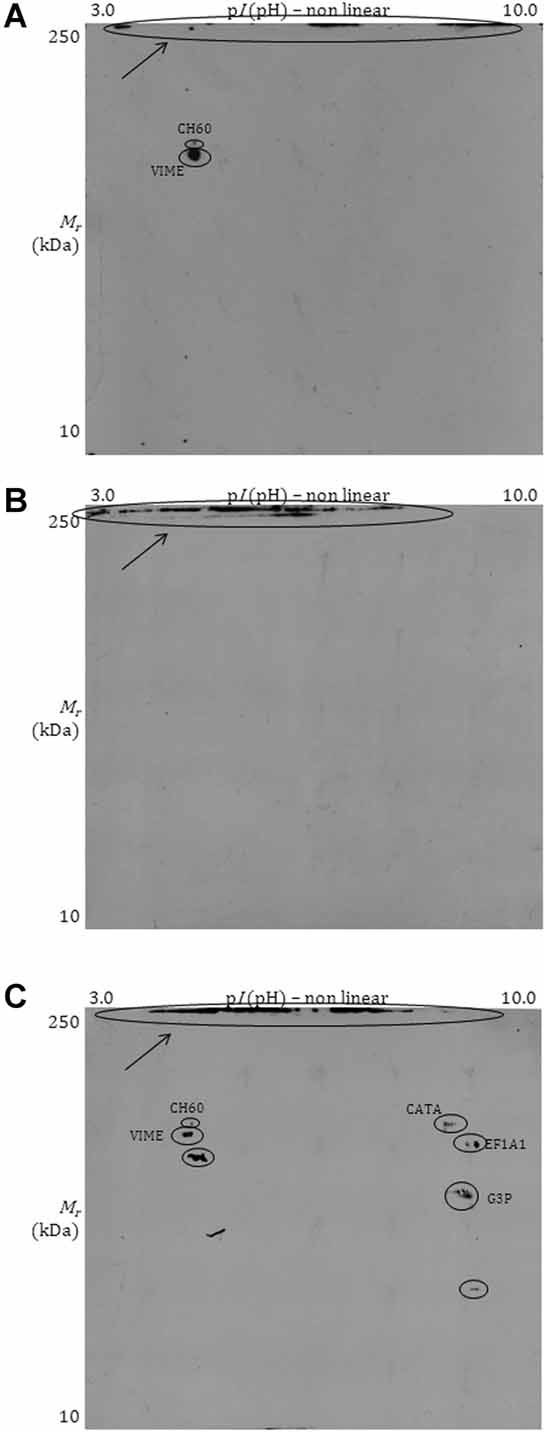
Redox proteomic analysis. Carbonylated proteins in non-AKU (A), “white” (B), and “black” (C) AKU chondrocytes are indicated with their abbreviated name. High molecular weight aggregates stacked on the top of the SDS–PAGE gel are highlighted with arrows. Representative images from a triplicate set are shown.

Differently from what observed with the comparative proteomic analysis, when we investigated protein oxidation through immunorevelation of carbonylated proteins we found that “white” and “black” AKU chondrocytes showed quite distinct patterns (Fig. 5). Gel-matching with the corresponding silver stained replica gel allowed the identification of almost all the spots immunorevealed under the conditions tested (Table II).

**Table II:** Redox proteomics of AKU chondrocytes

Spot	AN ^1^	Gene	Protein	Biological processes ^2^	CTR	CHW	CHB
Energy							
Carbohydrate metabolism							
G3P	P04406	*GAPDH*	Glyceraldehyde-3-phosphate dehydrogenase	Glycolysis, response to oxidative stress	**+**		**+**
Transcription, synthesis, and turnover of proteins							
EF1A1	P68104	*EEF1A1*	Elongation factor 1-alpha 1	Protein biosynthesis			**+**
Cellular organization							
Intermediate filaments family							
VIME	P08670	*VIM*	Vimentin	Structural constituent of cytoskeleton, cell motion	**+**		**+**
Cell rescue, defence, and stress							
CATA	P04040	*CAT*	Catalase	Response to oxidative stress			**+**
CH60	P10809	*HSP60*	60 kDa heat shock protein, mitochondrial	Protein folding and maturation, response to unfolded protein			**+**

Identification of carbonylated (+) proteins in non-AKU control (CTR), AKU “white” (CHW) and “black” (CHB) chondrocytes.

In control chondrocytes, we could identify as carbonylated only the proteins G3P (*energy*) and VIME (*cell organization*). In AKU cells, whereas “white” chondrocytes had no resolved immunoreactive spots but only high molecular weight protein aggregates stacked on the top of the SDS–PAGE gel, “black” AKU chondrocytes showed, together with carbonylated high molecular weight protein aggregates, the following carbonylated proteins: G3P (*energy*), EF1A1 (*transcription*, *synthesis*, and *turnover of proteins*), VIME (*cell organization*), CATA and CH60 (*cell rescue*, *defence*, and *stress*).

## Discussion

In this study we have provided, to the best of our knowledge, the first proteomic and redox proteomic characterization of ex vivo AKU chondrocytes as models of ochronotic arthropathy. Our main aim was to gain insights into those AKU-related pathophysiological processes that allow ochronosis to develop in connective tissues, especially joints. We were able to subculture two distinct populations, namely “white” and “black” AKU chondrocytes, on the basis of the macroscopic appearance of donors' hip ochronotic cartilage from which the cells were obtained. Both cell types, maintained in culture conditions resembling the original pathological ones thanks to the addition of HGA to the culture medium, were characterized by increased apoptosis, NO release, and levels of pro-inflammatory cytokines similarly to what observed in OA, a pathology that share some features with alkaptonuric arthropathy. Interestingly, both the cell types contained intracellular ochronotic pigments, suggesting that ochronosis might involve joint cells before than the surrounding ECM. Profound alterations in the levels of proteins involved in cell defence, protein folding, and cell organization were found in AKU ochronotic chondrocytes together with an increased post-translational oxidation of proteins and oxidized high molecular weight protein aggregates.

One of the main functions of articular cartilage is to provide a structure dissipating excessive loading and withstanding tension, compression, and shearing forces (Juang et al., 2010). Chondrocytes are crucial players in maintaining cartilage integrity by activating catabolic or anabolic processes in response to external stimuli. The catabolic program activated upon inflammation involves the production of proteases and the induction of apoptosis together with the suppression of matrix synthesis. Conversely, the anabolic program promotes the secretion of specific cytokines, protease inhibitors, the production of ECM and cell proliferation (Lotz et al., 1995). An imbalance towards an increase in catabolic activities is thought to be involved in the disruption of cartilage integrity leading to loss of joint functions (Blanco Garcia, 1999; Kim and Blanco, 2007). We have already demonstrated an inflammatory status in AKU (Selvi et al., 2000) and IL-6 and IL-8 over-expression in AKU chondrocytes (unpublished data). In this work, we confirmed our previous findings and also highlighted that levels of NO were increased in AKU ochronotic cells concomitantly with an enhanced release of pro-inflammatory cytokines (IL-1β, IL-6, IL-8, and TNFα). Altogether, our results suggest that pro-inflammatory cytokines and NO might play important roles in cartilage and joint tissue disruption in AKU patients during the ochronotic progression.

NO plays pro-inflammatory and destructive effects in cartilages and it has been related to a variety of mechanisms promoting cartilage catabolism (Henrotin et al., 2005). Cytokines such as TNFα and IL-1β are important mediators of inflammation and cartilage degradation observed in OA (Cillero-Pastor et al., 2010). Both have been shown to modulate proteins regulating cytoskeleton, tissue organization, and cell morphology of human chondrocytes (Cillero-Pastor et al., 2010) and to induce the expression of PRDX1 (Cillero-Pastor et al., 2010), whose main function is to amend oxidative stress imposed by hydrogen peroxide. Similar results were confirmed in this work thanks to the proteomic characterization of AKU cells. TNFα was also found to down-regulate the expression of G3P and LDBH (Cillero-Pastor et al., 2010). Concomitantly with an enhanced release of TNFα, we observed in both AKU “white” and “black” chondrocytes a decrease in the abundance of G3P, whereas LDBH was under-expressed only in “white” chondrocytes. LDB is down-regulated also in OA patients (Ruiz-Romero et al., 2008). This suggests a lowering of the glycolysis rate and ATP production possibly predisposing to cell apoptosis, like observed upon TNFα stress (Cillero-Pastor et al., 2010), a finding corroborated in our work by a lower expression of ATPB protein (which is involved in ATP production) in “black” chondrocytes.

Inhibition of metabolism-related proteins is a common feature of OA (Iliopoulos et al., 2010). Nevertheless, the over-expression of proteins with functions in energy and protein fate, such as G3P, PDIA1, HS47, and CALR, as a function of catabolic program was highlighted in a rat chondrocyte model exposed to pressure overload (Juang et al., 2010). Interestingly, in our work we found that G3P, PDIA1, and CALR were under-expressed in AKU ochronotic chondrocytes, probably pointing out to an impaired ability of cells to produce a cartilage structure optimally withstanding pressure forces. In this light, it is noteworthy to mention that load bearing joints are the first to show the deposition of ochronotic pigments (La Du, 1991).

IL-1β is a key mediator of inflammation and cartilage degradation in OA, but it also play a role in oxidative stress generation since it can induce an imbalance in the activity of fundamental anti-oxidant enzymes, such as superoxide dismutase, catalase, and glutathione peroxidase, as demonstrated recently in chondrocytes (Mathy-Hartert et al., 2008). Though to a lesser extent, also IL-6 was found to induce such a disregulation of antioxidant enzymes. These changes can ultimately lead to a transient accumulation of ROS (Mathy-Hartert et al., 2008) which might induce, in turn, oxidation of cellular constituents. On this basis, and also taking into account the HGA-induced oxidative post-translational modifications of proteins that we highlighted in different AKU models (Braconi et al., 2010a, b; Tinti et al., 2010, 2011b), we performed a redox proteomic analysis to evaluate protein carbonylation in AKU ochronotic cells. Notably, in this work we found responses that were similar to what previously showed in a chondrocytic cell line treated with 0.33 mM HGA and used as an in vitro model of AKU ochronosis (Braconi et al., 2010b). We confirmed the identity of proteins most likely to undergo HGA-induced oxidation and also the production of high molecular weight aggregates of oxidized proteins, providing in turn indirect evidences of the auto-oxidation of HGA into BQA, which is known to promote protein aggregation mediated by oxidative phenomena. Protein oxidation and aggregation might help, in vivo, the production of ochronotic pigments.

In conclusion, the findings presented in this work suggest that alkaptonuric ochronosis might be mediated by profound alterations in chondrocyte structure and organization leading to an impaired ability of cartilages to withstand loading forces. Concomitantly, pro-inflammatory stimuli and NO-mediated damage can contribute to cartilage degradation and chondrocyte apoptosis. AKU ochronotic cells are also characterized by a lower expression of proteins for the protection against stresses (leading to consequent protein oxidation, induced by HGA and its conversion into BQA, a spontaneous reaction which is an additional source of ROS). This, together with a lower expression of proteins promoting protein folding, points towards an increased protein aggregation which might lay the basis in vivo for the production and deposition of ochronotic pigments.

## Abbreviations

AKU: alkaptonuria
BQA: benzoquinone acetic acid
CHAPS: 3-[(3-cholamidopropyl)dimethylammonio]-1-propanesulfonate
DNPH: 2,4-dinitrophenylhydrazine
DTE: 1,4-dithioerythritol
HGA: homogentisic acid
IAA: iodoacetamide
IEF: isoelectric focusing
MS: mass spectrometry
PAGE: polyacrylamide gel electrophoresis
ROS: reactive oxygen species
SDS: sodium dodecyl sulfate
