# Supplementary material for: Biochemical and Proteomic Characterization of Alkaptonuric Chondrocytes
Source: J Cell Physiol. 2011 Dec 29;227(9):3333–43. doi: 10.1002/jcp.24033 (PMC3427902; doi:10.1002/jcp.24033)
Supplement: Supplementary file 1 [file jcp0227-3333-SD1.pdf]

Biochemical and proteomic characterization of alkaptonuric chondrocytes

Supplementary Material

Results of the comparative proteomic analysis on "white" and "black" AKU chondrocytes

The most relevant proteins that were found to be differentially expressed in 'white' and 'black' AKU chondrocytes with respect to non-AKU control chondrocytes are described here according to their functional classification.

Metabolism

Within this functional class, the under-expression of dihydropyrimidinase-related protein 2 (DPYL2) in 'white' chondrocytes was noteworthy. DPYL2 is one of the major proteins involved in remodeling of cell cytoskeleton and signal pathway (Inagaki et al., 2001); it is already known to be significantly under-expressed in OA chondrocyte mitochondria (Ruiz-Romero et al., 2009).

In both 'white' and 'black' chondrocytes, we also found an increased abundance of nucleoside diphosphate kinase A (NDKA); similarly, an increase in the isoform NDKB was observed in osteoarthritis (OA) chondrocyte mitochondria (Ruiz-Romero et al., 2009).

Energy

Chondrocytes in articular cartilage have a very limited blood supply (creating nutrient and oxygen gradients) and are likely to face low oxygen tensions. Whereas chondrocytes closest to the synovium are probably exposed to a normoxic tension, cells in the deepest regions of the cartilage may have to face lower oxygen tensions, and some authors suggested that the low rate of aerobic respiration in cartilage cells may be an adaptive response to such an environment (Marcus, 1973). Though cells in the more superficial zones of articular cartilage can rely on mitochondrial oxidative phosphorylation, cells facing anoxia use anaerobic glycolysis as a fundamental way to generate ATP in order to survive and produce ECM (Blanco et al., 2004; Johnson et al., 2004).

A proteomic study on how hypoxia differently modulates normal and OA chondrocytes revealed that OA cells are less reactive to an hypoxic *milieu*, showing important alterations in the abundance of proteins committed to energy production (Ruiz-Romero et al., 2010). Similarly, when we analyzed the expression of proteins involved in energy metabolism, we found an under-expression of glyceraldehyde-3-phosphate dehydrogenase (G3P). The under-expression of this protein was proposed by others as a possible biomarker of OA (Ruiz-Romero et al., 2008). Concomitantly, in 'black' chondrocytes we detected an increased expression of transketolase (TKT). TKT, belonging to the pentose-phosphate pathway, plays a key role in counteracting oxidative stress since it can provide the cells with reducing power. This finding might indicate that 'black' AKU cells experience oxidative stress.

Transcription, synthesis and turnover of proteins

In this functional class we found a general under-expression of identified proteins, including elongation factor 1-alpha 1 (EF1A1), elongation factor Tu (EFTU), and cathepsin D (CATD). CATD is the most abundant cellular endopeptidase in chondrocytes (Ruiz-Romero et al., 2005) and its role in bone metabolism and cartilage turnover was suggested (Handley et al., 2001). Notably, CATD contributes to the proteolytic processing of the core protein of aggrecan in the cartilage, though the potential importance of this enzyme in joint diseases, such as OA, appears relegated to intracellular catabolism (Nakase et al., 2000). In OA, chondrocytes have increased expression and activity of catabolic enzymes such as cathepsin B and D, and both enzymes together with cathepsin K and MMPs help the degradation of ECM. Nonetheless, a significant decrease in chondrocytes positive for CATD was shown in an in vivo model of OA, suggesting that loading forces may modulate such a phenomenon (Bowe et al., 2007) and that CATD may have distinct functions within articular cartilages. In our investigations, we found that levels of CATD were 4.7 and 3.8 times lower in 'white' and 'black' AKU chondrocytes, respectively.

Protein fate

Both 'white' and 'black' AKU chondrocytes showed a significant under expression of identified proteins involved in folding, maturation and transport of proteins. Particularly, lower levels of calreticulin (CALR), 75 kDa glucose-regulated protein (GRP75), protein DJ-1 (PARK7), proteasome activator complex subunit 1 (PSME1) were common to both 'white' and 'black' AKU chondrocytes. Conversely, prolyl 4-hydroxylase subunit alpha-1 (P4HA1) was specifically under-expressed in 'white' AKU chondrocytes whereas protein disulfide-isomerase (PDIA1), transitional endoplasmic reticulum ATPase (TERA) and ubiquitin-conjugating enzyme E2 K (UBE2K) were specifically under-expressed in 'black' AKU chondrocytes with respect to the control. Notably, CALR is required for assembly and quality control of proteins, while PDIA1 has a fundamental role in triple helix collagen formation. GRP75 is a mitochondrial protein with anti-apoptotic functions induced by several stresses, when it is expected to help protein folding (Wadhwa et al., 2002).

Cellular organization

Like in other cells types, in chondrocytes the cytoskeleton consists of microfilaments made of actin, microtubules made of tubulin and intermediate filaments made of a variety of subunits. Cytoskeleton, establishing an intimate connection with ECM, plays an important role in maintaining cell shape and functions (Kim et al., 2003). In our proteomic analysis, we observed that tubulin (TBB5), gelsolin (GELS), tropomyosin alpha-3 chain (TPM3) and WD repeat and FYVE domain-containing protein 1 (WDF1) were under-expressed only in 'black' chondrocytes in comparison to control chondrocytes. Furthermore, we found that annexin A4 (ANXA4) and A5 (ANXA5), vimentin (VIME), and galectin-3 (LEG3) were under-expressed in both 'white' and 'black' AKU chondrocytes.

Among these proteins, it is noteworthy to mention that VIME helps cells tolerate different types of stress allowing a quick cytoskeleton adaptation through sequential degradation and rearrangement processes, being the most sensitive sensor for mechanically-induced stress (Li et al., 2010). The mechanical role of VIME is also postulated basing on its higher expression in chondrocytes from load-bearing cartilages (Langelier et al., 2000) or regions of it undergoing more important deformation

stresses (Eggli et al., 1988). VIME is thus necessary to maintain the chondrocyte stiffness and allow a proper mechanotransduction; changes in VIME organization are considered to play a role in the pathophysiology of OA (in affected cartilages, it is under-expressed (Li et al., 2010; Ruiz-Romero et al., 2009)) and correlate with disease progression (Blain, 2009; Blain et al., 2006; Haudenschild et al., 2010; Lambrecht et al., 2008). In our investigations, VIME levels were 5.2 and 4.1 times lower in 'white' and 'black' AKU chondrocytes, respectively.

Furthermore, GELS regulates chondrocyte architecture and cell-matrix interactions. GELS was found to be lowered in synovial tissues from rheumatoid arthritis (Djouad et al., 2007) or upon IL-1β stress (Wilson et al., 2008) and associated with cytoskeletal remodelling in arthritis synovial cells (Aidinis et al., 2005; Vasilopoulos et al., 2007). We found that GELS levels were 2.0 times lower in 'black' AKU chondrocytes than in the control.

Cell rescue, defence and stress

Like previously indicated by our group in a chondrocytic line used as a model of AKU (Braconi et al., 2010b), levels of proteins involved in cell rescue and defence from stress showed the most interesting alterations. Oxidative stress plays an essential role in the development and progression of OA (Blanco et al., 2004; Gobezie et al., 2007; Johnson et al., 2004) and protection from oxidative stress is fundamental for the avascular tissue of cartilage and highly specialized tissues of joints (Gobezie et al., 2007). Possible relationships among ageing, chronic inflammation and cartilage degradation in OA were reviewed recently (Henrotin et al., 2007); nevertheless, the pathology-related antioxidant status of chondrocytes need to be addressed in greater detail (Henrotin and Kurz, 2007). Importantly, ROS are known to be implicated in the pathogenesis of AKU, like we demonstrated previously in several in vitro models (Braconi et al., 2010a; Braconi et al., 2010b; Tinti et al., 2010).

In this work, we found that AKU chondrocytes showed altered levels of proteins fundamental for the protection from oxidative stress. Lower protein levels were found for the mitochondrial 60 kDa HSP, (CH60), alpha-crystallin B chain (CRYAB), endoplasmin (ENPL), glutathione S-transferase omega-1 (GSTO1), glutathione S-transferase P (GSTP1), heat shock 70 kDa protein 4 (HSP74) and mitochondrial superoxide dismutase (SODM). On the contrary, alpha-crystallin A chain (CRYAA), peroxiredoxin-1

(PRDX1), peroxiredoxin-6 (PRDX6) and serpin H1 (SERPH) were over-expressed both in 'white' and 'black' AKU chondrocytes compared to the control. Notably, PRDX1 is among the proteins released by chondrocyes upon IL-1 stimulation (Catterall et al., 2006), while SODM is a key factor in controlling chondrocyte metabolism and redox-status (De Ceuninck, 2008; Ruiz-Romero et al., 2009). SODM, dismutating peroxide anions, can protect the cells from oxidative damages but lower levels of SODM characterize OA cartilages (Matsumoto et al., 1991; Tiku et al., 2000). In the present work, we showed an important deficiency of SODM in 'white' (-5.7 fold-change) and 'black (-4.6 fold-change) AKU chondrocytes. A similar trend was shown by glutathione S-transferases (GSTs), which have protective roles during oxidative stress (Higuchi, 2004; Vaillancourt et al., 2008) but are lowered in OA (Carlo and Loeser, 2003). GSTO1 has been already addressed as a mediator of inflammation in OA, and several studies associated GSTO1 to stress-induced apoptosis or human pathologies such as Alzheimer's and Parkinson's disease (Ruiz-Laguna et al., 2005) besides OA, where it has been indicated as a disease marker (Ruiz-Romero et al., 2008). GSTs are very important detoxifying enzymes: they can react with and neutralize ROS but also quinones (Higuchi, 2004). This, together with the above-mentioned SODM deficiency, reinforce the hypothesis that AKU cells cannot adequately control ROS- and quinone-mediated toxicity.

Levels of heat shock 70 kDa protein 4 (HSP74) correlates with OA clinical severity (Kubo et al., 1985; Takahashi et al., 1997). Additionally, NO-induced activation of HSPs is thought to suppress apoptosis (Mosser et al., 2000; Terauchi et al., 2003). In our proteomic analysis, we showed a significant under-expression of HSP74 in 'white' (-2.8 fold-change) and 'black' (-5.4 fold-change) chondrocytes with respect to control, suggesting that the lack of this protein may promote NO-induced apoptosis induced AKU chondrocytes.

References

Aidinis V, Carninci P, Armaka M, Witke W, Harokopos V, Pavelka N, Koczan D, Argyropoulos C, Thwin MM, Moller S, Waki K, Gopalakrishnakone P, Ricciardi-Castagnoli P, Thiesen HJ, Hayashizaki Y,

Kollias G. 2005. Cytoskeletal rearrangements in synovial fibroblasts as a novel pathophysiological determinant of modeled rheumatoid arthritis. PLoS Genet 1(4):e48.

Blain EJ. 2009. Involvement of the cytoskeletal elements in articular cartilage homeostasis and pathology. Int J Exp Pathol 90(1):1-15.

Blain EJ, Gilbert SJ, Hayes AJ, Duance VC. 2006. Disassembly of the vimentin cytoskeleton disrupts articular cartilage chondrocyte homeostasis. Matrix Biol 25(7):398-408.

Blanco FJ, Lopez-Armada MJ, Maneiro E. 2004. Mitochondrial dysfunction in osteoarthritis. Mitochondrion 4(5-6):715-728.

Bowe EA, Murray RC, Jeffcott LB, Davies ME. 2007. Do the matrix degrading enzymes cathepsins B and D increase following a high intensity exercise regime? Osteoarthritis Cartilage 15(3):343-349.

Braconi D, Laschi M, Amato L, Bernardini G, Millucci L, Marcolongo R, Cavallo G, Spreafico A, Santucci A. 2010a. Evaluation of anti-oxidant treatments in an in vitro model of alkaptonuric ochronosis. Rheumatology (Oxford) 49(10):1975-1983.

Braconi D, Laschi M, Taylor AM, Bernardini G, Spreafico A, Tinti L, Gallagher JA, Santucci A. 2010b. Proteomic and redox-proteomic evaluation of homogentisic acid and ascorbic acid effects on human articular chondrocytes. J Cell Biochem 111(4):922-932.

Carlo MD, Jr., Loeser RF. 2003. Increased oxidative stress with aging reduces chondrocyte survival: correlation with intracellular glutathione levels. Arthritis Rheum 48(12):3419-3430.

Catterall JB, Rowan AD, Sarsfield S, Saklatvala J, Wait R, Cawston TE. 2006. Development of a novel 2D proteomics approach for the identification of proteins secreted by primary chondrocytes after stimulation by IL-1 and oncostatin M. Rheumatology (Oxford) 45(9):1101-1109.

De Ceuninck F. 2008. The application of proteomics to articular cartilage: a new hope for the treatment of osteoarthritis. Joint Bone Spine 75(4):376-378.

Djouad F, Delorme B, Maurice M, Bony C, Apparailly F, Louis-Plence P, Canovas F, Charbord P, Noel D, Jorgensen C. 2007. Microenvironmental changes during differentiation of mesenchymal stem cells towards chondrocytes. Arthritis Res Ther 9(2):R33.

Eggli PS, Hunziker EB, Schenk RK. 1988. Quantitation of structural features characterizing weight- and less-weight-bearing regions in articular cartilage: a stereological analysis of medial femoral condyles in young adult rabbits. Anat Rec 222(3):217-227.

Gobezie R, Kho A, Krastins B, Sarracino DA, Thornhill TS, Chase M, Millett PJ, Lee DM. 2007. High abundance synovial fluid proteome: distinct profiles in health and osteoarthritis. Arthritis Res Ther 9(2):R36.

Handley CJ, Mok MT, Ilic MZ, Adcocks C, Buttle DJ, Robinson HC. 2001. Cathepsin D cleaves aggrecan at unique sites within the interglobular domain and chondroitin sulfate attachment regions that are also cleaved when cartilage is maintained at acid pH. Matrix Biol 20(8):543-553.

Haudenschild DR, Chen J, Pang N, Steklov N, Grogan SP, Lotz MK, D'Lima DD. 2010. Vimentin contributes to changes in chondrocyte stiffness in osteoarthritis. J Orthop Res 29(1):20-25.

Henrotin Y, Blanco FJ, Aigner T, Kurz B. 2007. The significance of oxidative stress in articular cartilage ageing and degradation. Current Rheumatology Reviews 3:261-274.

Henrotin Y, Kurz B. 2007. Antioxidant to treat osteoarthritis: dream or reality? Curr Drug Targets 8(2):347-357.

Higuchi Y. 2004. Glutathione depletion-induced chromosomal DNA fragmentation associated with apoptosis and necrosis. J Cell Mol Med 8(4):455-464.

Inagaki N, Chihara K, Arimura N, Menager C, Kawano Y, Matsuo N, Nishimura T, Amano M, Kaibuchi K. 2001. CRMP-2 induces axons in cultured hippocampal neurons. Nat Neurosci 4(8):781-782.

Johnson K, Svensson CI, Etten DV, Ghosh SS, Murphy AN, Powell HC, Terkeltaub R. 2004. Mediation of spontaneous knee osteoarthritis by progressive chondrocyte ATP depletion in Hartley guinea pigs. Arthritis Rheum 50(4):1216-1225.

Kim SJ, Hwang SG, Kim IC, Chun JS. 2003. Actin cytoskeletal architecture regulates nitric oxide-induced apoptosis, dedifferentiation, and cyclooxygenase-2 expression in articular chondrocytes via mitogen-activated protein kinase and protein kinase C pathways. J Biol Chem 278(43):42448-42456.

Kubo T, Towle CA, Mankin HJ, Treadwell BV. 1985. Stress-induced proteins in chondrocytes from patients with osteoarthritis. Arthritis Rheum 28(10):1140-1145.

Lambrecht S, Verbruggen G, Verdonk PC, Elewaut D, Deforce D. 2008. Differential proteome analysis of normal and osteoarthritic chondrocytes reveals distortion of vimentin network in osteoarthritis. Osteoarthritis Cartilage 16(2):163-173.

Langelier E, Suetterlin R, Hoemann CD, Aebi U, Buschmann MD. 2000. The chondrocyte cytoskeleton in mature articular cartilage: structure and distribution of actin, tubulin, and vimentin filaments. J Histochem Cytochem 48(10):1307-1320.

Li H, Yang HS, Wu TJ, Zhang XY, Jiang WH, Ma QL, Chen YX, Xu Y, Li S, Hua ZC. 2010. Proteomic analysis of early-response to mechanical stress in neonatal rat mandibular condylar chondrocytes. J Cell Physiol 223(3):610-622.

Marcus RE. 1973. The effect of low oxygen concentration on growth, glycolysis, and sulfate incorporation by articular chondrocytes in monolayer culture. Arthritis Rheum 16(5):646-656.

Matsumoto H, Silverton SF, Debolt K, Shapiro IM. 1991. Superoxide dismutase and catalase activities in the growth cartilage: relationship between oxidoreductase activity and chondrocyte maturation. J Bone Miner Res 6(6):569-574.

Mosser DD, Caron AW, Bourget L, Meriin AB, Sherman MY, Morimoto RI, Massie B. 2000. The chaperone function of hsp70 is required for protection against stress-induced apoptosis. Mol Cell Biol 20(19):7146-7159.

Nakase T, Kaneko M, Tomita T, Myoui A, Ariga K, Sugamoto K, Uchiyama Y, Ochi T, Yoshikawa H. 2000. Immunohistochemical detection of cathepsin D, K, and L in the process of endochondral ossification in the human. Histochem Cell Biol 114(1):21-27.

Ruiz-Laguna J, Abril N, Prieto-Alamo MJ, Lopez-Barea J, Pueyo C. 2005. Tissue, species, and environmental differences in absolute quantities of murine mRNAs coding for alpha, mu, omega, pi, and theta glutathione S-transferases. Gene Expr 12(3):165-176.

Ruiz-Romero C, Calamia V, Mateos J, Carreira V, Martinez-Gomariz M, Fernandez M, Blanco FJ. 2009. Mitochondrial dysregulation of osteoarthritic human articular chondrocytes analyzed by proteomics: a decrease in mitochondrial superoxide dismutase points to a redox imbalance. Mol Cell Proteomics 8(1):172-189.

Ruiz-Romero C, Calamia V, Rocha B, Mateos J, Fernandez-Puente P, Blanco FJ. 2010. Hypoxia conditions differentially modulate human normal and osteoarthritic chondrocyte proteomes. J Proteome Res 9(6):3035-3045.

Ruiz-Romero C, Carreira V, Rego I, Remeseiro S, Lopez-Armada MJ, Blanco FJ. 2008. Proteomic analysis of human osteoarthritic chondrocytes reveals protein changes in stress and glycolysis. Proteomics 8(3):495-507.

Ruiz-Romero C, Lopez-Armada MJ, Blanco FJ. 2005. Proteomic characterization of human normal articular chondrocytes: a novel tool for the study of osteoarthritis and other rheumatic diseases. Proteomics 5(12):3048-3059.

Takahashi K, Kubo T, Arai Y, Imanishi J, Kawata M, Hirasawa Y. 1997. Localization of heat shock protein in osteoarthritic cartilage. Scand J Rheumatol 26(5):368-375.

Terauchi R, Takahashi KA, Arai Y, Ikeda T, Ohashi S, Imanishi J, Mazda O, Kubo T. 2003. Hsp70 prevents nitric oxide-induced apoptosis in articular chondrocytes. Arthritis Rheum 48(6):1562-1568.

Tiku ML, Shah R, Allison GT. 2000. Evidence linking chondrocyte lipid peroxidation to cartilage matrix protein degradation. Possible role in cartilage aging and the pathogenesis of osteoarthritis. J Biol Chem 275(26):20069-20076.

Tinti L, Spreafico A, Braconi D, Millucci L, Bernardini G, Chellini F, Cavallo G, Selvi E, Galeazzi M, Marcolongo R, Gallagher JA, Santucci A. 2010. Evaluation of antioxidant drugs for the treatment of ochronotic alkaptonuria in an in vitro human cell model. J Cell Physiol 225(1):84-91.

Vaillancourt F, Fahmi H, Shi Q, Lavigne P, Ranger P, Fernandes JC, Benderdour M. 2008. 4-Hydroxynonenal induces apoptosis in human osteoarthritic chondrocytes: the protective role of glutathione-S-transferase. Arthritis Res Ther 10(5):R107.

Vasilopoulos Y, Gkretsi V, Armaka M, Aidinis V, Kollias G. 2007. Actin cytoskeleton dynamics linked to synovial fibroblast activation as a novel pathogenic principle in TNF-driven arthritis. Ann Rheum Dis 66 Suppl 3:iii23-28.

Wadhwa R, Taira K, Kaul SC. 2002. An Hsp70 family chaperone, mortalin/mthsp70/PBP74/Grp75: what, when, and where? Cell Stress Chaperones 7(3):309-316.

Wilson R, Belluoccio D, Little CB, Fosang AJ, Bateman JF. 2008. Proteomic characterization of mouse cartilage degradation in vitro. Arthritis Rheum 58(10):3120-3131.
